# (2,7-Di­meth­oxy­naphthalen-1-yl)(naph­thalen-1-yl)methanone

**DOI:** 10.1107/S1600536813008854

**Published:** 2013-04-05

**Authors:** Takehiro Tsumuki, Atsumi Isogai, Hiroyuki Kawano, Noriyuki Yonezawa, Akiko Okamoto

**Affiliations:** a Department of Organic and Polymer Materials Chemistry, Tokyo University of Agriculture & Technology, Koganei, Tokyo 184-8588, Japan; b Division of Liberal Arts, Kogakuin University, Hachioji, Tokyo 192-0015, Japan

## Abstract

The asymmetric unit of the title compound, C_23_H_18_O_3_, contains two independent mol­ecules (*A* and *B*). Each mol­ecule has essentially the same conformation (r.m.s. deviation of fitted mol­ecules = 0.173 Å) with the aromatic rings twisted in a near perpendicular manner. The dihedral angles between the two naphthalene ring systems are 79.07 (4) and 88.19 (4)° in the two independent mol­ecules. In the crystal, the *A* mol­ecules are connected by C—H⋯O inter­actions, forming chains along the *b*-axis direction. Further C—H⋯O inter­actions between the H atoms of the meth­oxy group and the O atoms of the carbonyl units link the *A* and *B* mol­ecules, forming a three-dimensional network.

## Related literature

For electrophilic aroylation of naphthalene derivatives, see: Okamoto & Yonezawa (2009 ▶); Okamoto *et al.* (2011 ▶). For the structures of closely related compounds, see: Nakaema *et al.* (2008 ▶); Kato *et al.* (2010 ▶); Tsumuki *et al.* (2012 ▶, 2013 ▶); Sasagawa *et al.* (2013 ▶).
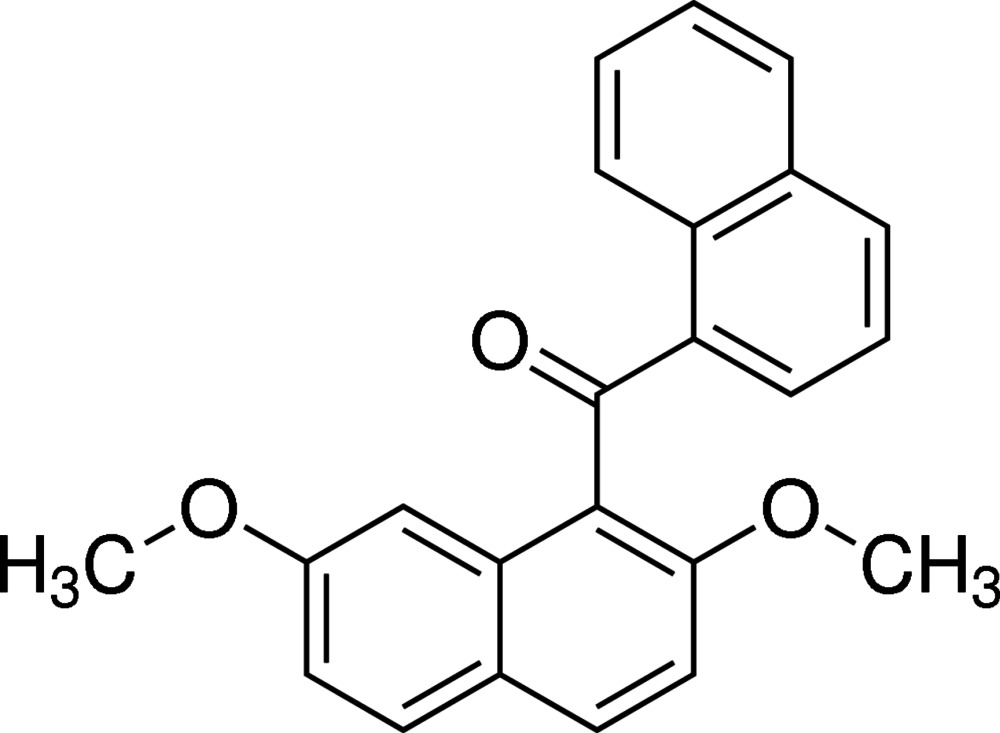


## Experimental

### 

#### Crystal data

C_23_H_18_O_3_
*M* *_r_* = 342.37
Monoclinic, 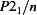

*a* = 16.1451 (3) Å
*b* = 7.51303 (14) Å
*c* = 29.0107 (5) Å
β = 98.547 (1)°
*V* = 3479.88 (11) Å^3^
*Z* = 8
Cu *K*α radiation
μ = 0.69 mm^−1^
*T* = 193 K
0.50 × 0.20 × 0.10 mm

#### Data collection

Rigaku R-AXIS RAPID diffractometer
Absorption correction: numerical (*NUMABS*; Higashi, 1999 ▶) *T*_min_ = 0.725, *T*_max_ = 0.934
62016 measured reflections
6349 independent reflections
5490 reflections with *I* > 2σ(*I*)
*R*_int_ = 0.032

#### Refinement

*R*[*F* ^2^ > 2σ(*F* ^2^)] = 0.037
*wR*(*F* ^2^) = 0.105
*S* = 1.07
6349 reflections
470 parameters
H-atom parameters constrained
Δρ_max_ = 0.21 e Å^−3^
Δρ_min_ = −0.24 e Å^−3^

### 

Data collection: *PROCESS-AUTO* (Rigaku, 1998 ▶); cell refinement: *PROCESS-AUTO*; data reduction: *PROCESS-AUTO*; program(s) used to solve structure: *Il Milione* (Burla *et al.*, 2007 ▶); program(s) used to refine structure: *SHELXL97* (Sheldrick, 2008 ▶); molecular graphics: *ORTEPIII* (Burnett & Johnson, 1996 ▶); software used to prepare material for publication: *SHELXL97*.

## Supplementary Material

Crystal structure: contains datablock(s) I, global. DOI: 10.1107/S1600536813008854/vm2191sup1.cif

Structure factors: contains datablock(s) I. DOI: 10.1107/S1600536813008854/vm2191Isup2.hkl

Supplementary material file. DOI: 10.1107/S1600536813008854/vm2191Isup3.cml

Additional supplementary materials:  crystallographic information; 3D view; checkCIF report

## Figures and Tables

**Table 1:** Hydrogen-bond geometry (Å, °)

*D*—H⋯*A*	*D*—H	H⋯*A*	*D*⋯*A*	*D*—H⋯*A*
C12—H12*B*⋯O6	0.98	2.51	3.2862 (18)	136
C19—H19⋯O3^i^	0.95	2.40	3.2418 (17)	148
C15—H15⋯O3	0.95	2.19	2.8397 (16)	125
C38—H38⋯O6	0.95	2.25	2.8548 (17)	121

Symmetry code: (i) 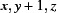.

**Fig. 1.**
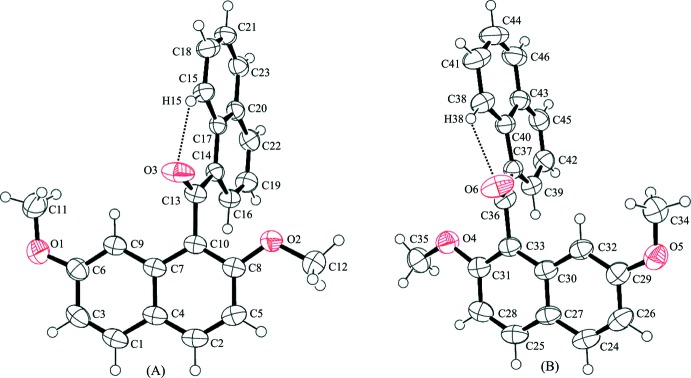
The structure of the two independent molecules (A) and (B), with atom numbering. Displacement ellipsoids are drawn at the 50% probability level. The intramolecular C—H···O interaction is shown as a dashed line (see Table 1 for details).

**Fig. 2.**
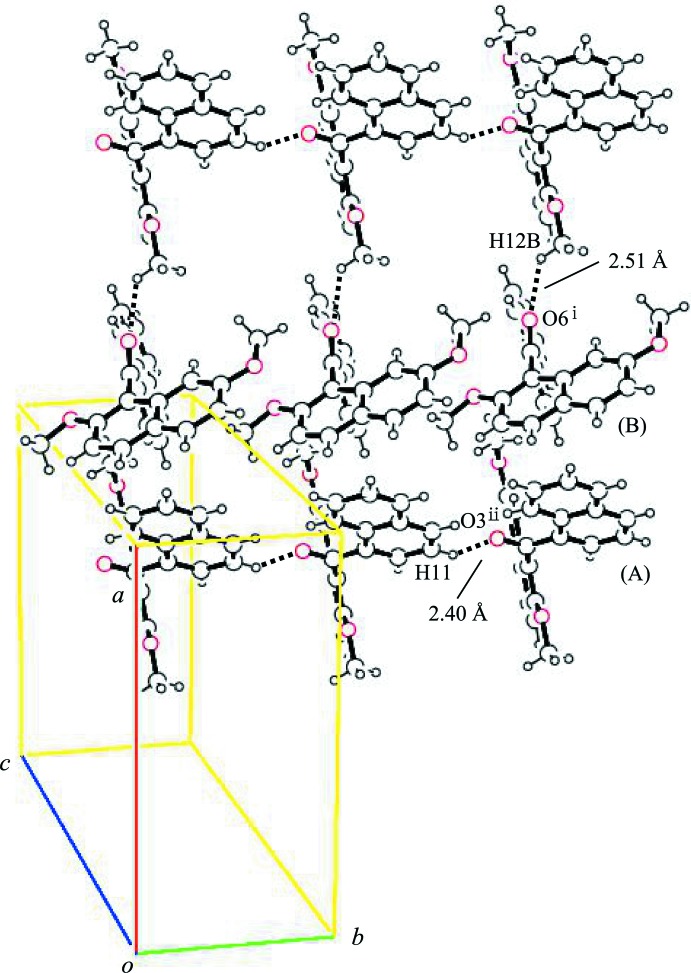
A partial view of the crystal packing of the title compound, showing the intermolecular C—H···O interactions (see Table 1 for details; symmetry code: (i) x, 1 + y, z).

## supplementary crystallographic information

### Comment

In the course of our study on selective electrophilic aromatic aroylation of the naphthalene ring core, 1,8-diaroylnaphthalene compounds have proved to be formed regioselectively by the aid of a suitable acidic mediator (Okamoto & Yonezawa, 2009, Okamoto *et al.*, 2011). Recently, we have reported the crystal structures of several 1,8-diaroylated naphthalene homologues exemplified by 1,8-dibenzoyl-2,7-dimethoxynaphthalene (Nakaema *et al.*, 2008), 2,7-diethoxy-1,8-bis(1-naphthoyl)naphthalene [{2,7-diethoxy-8-[(naphthalen-1-yl)carbonyl]naphthalen-1-yl}(naphthalen-1-yl)methanone; Tsumuki *et al.*, 2013]. The aroyl groups at the 1,8-positions of the naphthalene rings in these compounds are connected almost perpendicularly and oriented in opposite directions. Moreover, we have reported crystal structures of 1-monoaroylnapthalene compounds such as 1-benzoyl-2,7-dimethoxynaphthalene [(2,7-dimethoxynaphthalen-1-yl)(phenyl)methanone; Kato *et al.*, 2010], 2,7-dimethoxy-1-(2-naphthoyl)naphthalene (Tsumuki *et al.*, 2012), and 1-(4-methoxybenzoyl)-2,7-dimethoxynaphthalene [(2,7-dimethoxynaphthalen-1-yl)(4-methoxyphenyl)methanone; Sasagawa *et al*. 2013]. They have essentially the same non-coplanarly accumulated aromatic-rings structure as the homologous 1,8-diaroylnaphthalenes. As a part of our ongoing studies on the molecular structures of these kinds of homologous molecules, the X-ray crystal structure of the title compound, the 2,7-dimethoxynaphthalene bearing an *α*-naphthoyl group at the 1-position, is discussed in this article.

There are two independent molecules in the crystal structure of the title compound. The independent molecules are labeled (A) and (B) and show intramolecular C—H···O interactions between the carbonyl oxygen atoms and hydrogen atoms of the naphthoyl groups (Fig. 1 and Table 1). Each independent molecule has essentially the same non-coplanar structure as indicated by a least-squares fit of both molecules (r.m.s. deviation 0.173 Å). The naphthalene ring of the naphthoyl group and 2,7-dimethoxynaphthalene ring in molecules (A) and (B) make similar dihedral angles with each other and torsion angles with the ketonic carbonyl moieties. The differences of the dihedral angles and the torsion angles between molecules (A) and (B) are smaller than 10°. The respective dihedral angles between the best plane of the two naphthalene rings in molecules (A) and (B) are 79.07 (4)° and 88.19 (4)°. The torsion angles between the bridging carbonyl moieties and the 2,7-dimethoxynaphthalene unit in molecules (A) and (B) are 73.42 (16)° (C7—C10—C13—O3) and -68.22 (18)° (C30—C33—C36—O6). On the other hand, the torsion angles between the bridging carbonyl moieties and the naphthalene rings of naphthoyl groups in molecules (A) and (B) are rather small [O3—C13—C14—C17 torsion angle = 3.46 (19)° for molecule (A)] and [O6—C36—C37—C40 torsion angle = -11.4 (2)° for molecule (B)].

In the molecular packing, the molecules (A) are linked into chains along the *b* axis direction by C—H···O interactions between the naphthoyl groups. Both molecules (A) and (B) are connected by C—H···O interactions between the hydrogen atoms of the methoxy group and the oxygen atoms of the carbonyl moieties, forming a three-dimensional network (Fig.2, Table 1).

### Experimental

To a solution of 1-naphthoyl chloride (419 mg, 2.2 mmol), AlCl_3_ (440 mg, 3.3 mmol) and CH_2_Cl_2_ (10 ml), 2,7-dimethoxynaphthalene (376 mg, 2.0 mmol) was added. The reaction mixture was stirred at 273 K for 6 h, then poured into ice-cold water. The aqueous layer was extracted with CHCl_3_ (20 ml × 3) and the combined extracts were washed with 2 *M* aqueous NaOH (20 ml × 3) followed by washing with brine (20 ml × 3). The organic layer thus obtained was dried over anhydrous MgSO_4_. The solvent was removed under reduced pressure to give a cake (yield 96%). The crude product was purified by recrystallization from hexane (isolated yield 65%). Yellow platelet single crystals suitable for X-ray diffraction were obtained by repeated crystallization from hexane. Spectroscopic data for the title compound are available in the archived CIF.

### Refinement

All the H atoms were located in a difference Fourier map and were subsequently refined as riding atoms: C—H = 0.95 (aromatic) and 0.98 (methyl) Å with *U*iso(H) = 1.2 *U*eq(C).

### Crystal data

**Table:**

C_23_H_18_O_3_	*F*(000) = 1440
*M**_r_* = 342.37	*D*_x_ = 1.307 Mg m^−^^3^
Monoclinic, *P*2_1_/*n*	Melting point = 391.4–392.7 K
Hall symbol: -P 2yn	Cu *K*α radiation, λ = 1.54187 Å
*a* = 16.1451 (3) Å	Cell parameters from 51750 reflections
*b* = 7.51303 (14) Å	θ = 3.0–68.2°
*c* = 29.0107 (5) Å	µ = 0.69 mm^−^^1^
β = 98.547 (1)°	*T* = 193 K
*V* = 3479.88 (11) Å^3^	Platelet, yellow
*Z* = 8	0.50 × 0.20 × 0.10 mm

### Data collection

**Table:**

Rigaku R-AXIS RAPID diffractometer	6349 independent reflections
Radiation source: fine-focus sealed tube	5490 reflections with *I* > 2σ(*I*)
Graphite monochromator	*R*_int_ = 0.032
Detector resolution: 10.000 pixels mm^-1^	θ_max_ = 68.2°, θ_min_ = 3.0°
ω scans	*h* = −19→19
Absorption correction: numerical (*NUMABS*; Higashi, 1999)	*k* = −8→8
*T*_min_ = 0.725, *T*_max_ = 0.934	*l* = −34→34
62016 measured reflections	

### Refinement

**Table:**

Refinement on *F*^2^	Secondary atom site location: difference Fourier map
Least-squares matrix: full	Hydrogen site location: inferred from neighbouring sites
*R*[*F*^2^ > 2σ(*F*^2^)] = 0.037	H-atom parameters constrained
*wR*(*F*^2^) = 0.105	*w* = 1/[σ^2^(*F*_o_^2^) + (0.0555*P*)^2^ + 0.6272*P*] where *P* = (*F*_o_^2^ + 2*F*_c_^2^)/3
*S* = 1.07	(Δ/σ)_max_ = 0.001
6349 reflections	Δρ_max_ = 0.21 e Å^−^^3^
470 parameters	Δρ_min_ = −0.24 e Å^−^^3^
0 restraints	Extinction correction: *SHELXL97* (Sheldrick, 2008), Fc^*^=kFc[1+0.001xFc^2^λ^3^/sin(2θ)]^-1/4^
Primary atom site location: structure-invariant direct methods	Extinction coefficient: 0.00173 (12)

### Special details

**Table:**

Experimental. Spectroscopic data for the title compound: ^1^H NMR δ (500 MHz, CDCl_3_): 3.67 (3*H*, s), 3.68 (3*H*, s), 7.02–7.05 (2*H*, m), 7.14 (1*H*, dd, *J* = 1.2, 8.6 Hz), 7.34 (1*H*, t, *J* = 8.6 Hz), 7.58–7.61 (2*H*, m), 7.69–7.74 (2*H*, m), 7.88 (1*H*, d, *J* = 8.6 Hz), 7.93 (1*H*, d, *J* = 8.6 Hz), 8.00 (1*H*, d, *J* = 8.6 Hz), 9.14 (1*H*, d, *J* = 8.6 Hz) p.p.m.; ^13^C NMR δ (125 MHz, CDCl_3_): 55.15, 56.41, 102.10, 110.57, 117.19, 123.94, 124.39,124.50, 126.17, 126.42, 128.28, 128.42, 129.61, 130.71, 131.20, 131.27, 132.14,133.39, 133.96, 136.20, 155.53, 159.08, 200.04 p.p.m.; IR (KBr): 1652, 1623,1510, 1250, 1227 cm^-1^; HRMS (*m/z*): [*M*+H]^+^ calcd. for C_23_H_19_O_3_, 343.1334, found, 343.1310.
Geometry. All s.u.'s (except the s.u. in the dihedral angle between two l.s. planes) are estimated using the full covariance matrix. The cell s.u.'s are taken into account individually in the estimation of s.u.'s in distances, angles and torsion angles; correlations between s.u.'s in cell parameters are only used when they are defined by crystal symmetry. An approximate (isotropic) treatment of cell s.u.'s is used for estimating s.u.'s involving l.s. planes.
Refinement. Refinement of *F*^2^ against ALL reflections. The weighted *R*-factor *wR* and goodness of fit *S* are based on *F*^2^, conventional *R*-factors *R* are based on *F*, with *F* set to zero for negative *F*^2^. The threshold expression of *F*^2^ > 2σ(*F*^2^) is used only for calculating *R*-factors(gt) *etc*. and is not relevant to the choice of reflections for refinement. *R*-factors based on *F*^2^ are statistically about twice as large as those based on *F*, and *R*- factors based on ALL data will be even larger.

### Fractional atomic coordinates and isotropic or equivalent isotropic displacement parameters (Å^2^)

**Table:**

	*x*	*y*	*z*	*U*_iso_*/*U*_eq_	
O1	1.01615 (7)	0.12392 (16)	0.33256 (3)	0.0616 (3)	
O2	0.70559 (6)	0.15461 (14)	0.12536 (3)	0.0508 (2)	
O3	0.89345 (7)	−0.05216 (13)	0.15993 (4)	0.0622 (3)	
O4	0.26783 (6)	−0.25199 (14)	0.10328 (3)	0.0554 (3)	
O5	0.40252 (6)	0.56893 (14)	0.00664 (3)	0.0569 (3)	
O6	0.43705 (6)	0.04372 (18)	0.12626 (3)	0.0639 (3)	
C1	0.79575 (10)	0.21648 (19)	0.31798 (5)	0.0494 (3)	
H1	0.7553	0.2445	0.3375	0.059*	
C2	0.68602 (9)	0.21983 (18)	0.24859 (5)	0.0462 (3)	
H2	0.6449	0.2450	0.2679	0.055*	
C3	0.87670 (10)	0.1930 (2)	0.33734 (5)	0.0529 (4)	
H3	0.8925	0.2048	0.3701	0.063*	
C4	0.76993 (8)	0.20020 (17)	0.26918 (4)	0.0421 (3)	
C5	0.66176 (8)	0.20393 (18)	0.20164 (5)	0.0467 (3)	
H5	0.6045	0.2154	0.1886	0.056*	
C6	0.93771 (9)	0.15083 (18)	0.30871 (5)	0.0471 (3)	
C7	0.83205 (8)	0.16239 (16)	0.24039 (4)	0.0382 (3)	
C8	0.72308 (8)	0.17014 (17)	0.17282 (4)	0.0411 (3)	
C9	0.91635 (8)	0.13863 (17)	0.26125 (4)	0.0417 (3)	
H9	0.9582	0.1142	0.2423	0.050*	
C10	0.80601 (8)	0.14999 (16)	0.19160 (4)	0.0379 (3)	
C11	1.08006 (10)	0.0764 (3)	0.30625 (6)	0.0700 (5)	
H11A	1.0859	0.1701	0.2834	0.084*	
H11B	1.0653	−0.0360	0.2899	0.084*	
H11C	1.1332	0.0624	0.3272	0.084*	
C12	0.62104 (9)	0.1748 (2)	0.10372 (5)	0.0599 (4)	
H12A	0.5994	0.2897	0.1126	0.072*	
H12B	0.5870	0.0786	0.1139	0.072*	
H12C	0.6185	0.1699	0.0698	0.072*	
C13	0.86907 (8)	0.10129 (17)	0.16010 (4)	0.0390 (3)	
C14	0.90082 (7)	0.24390 (16)	0.13158 (4)	0.0347 (3)	
C15	0.99264 (7)	0.04222 (18)	0.09065 (4)	0.0409 (3)	
H15	0.9762	−0.0593	0.1066	0.049*	
C16	0.87278 (8)	0.41462 (17)	0.13703 (4)	0.0409 (3)	
H16	0.8343	0.4354	0.1583	0.049*	
C17	0.95957 (7)	0.21113 (17)	0.09948 (4)	0.0345 (3)	
C18	1.04813 (8)	0.0228 (2)	0.05938 (5)	0.0472 (3)	
H18	1.0688	−0.0924	0.0537	0.057*	
C19	0.89927 (9)	0.55883 (18)	0.11217 (5)	0.0468 (3)	
H19	0.8787	0.6751	0.1166	0.056*	
C20	0.98600 (7)	0.35935 (17)	0.07484 (4)	0.0383 (3)	
C21	1.07479 (8)	0.1691 (2)	0.03572 (4)	0.0468 (3)	
H21	1.1138	0.1539	0.0145	0.056*	
C22	0.95445 (8)	0.53152 (18)	0.08181 (5)	0.0454 (3)	
H22	0.9721	0.6295	0.0650	0.055*	
C23	1.04435 (8)	0.33343 (19)	0.04336 (4)	0.0441 (3)	
H23	1.0625	0.4330	0.0273	0.053*	
C24	0.28062 (9)	0.2044 (2)	−0.04715 (5)	0.0553 (4)	
H24	0.2535	0.1747	−0.0775	0.066*	
C25	0.23599 (8)	−0.0818 (2)	−0.01668 (5)	0.0514 (4)	
H25	0.2100	−0.1140	−0.0471	0.062*	
C26	0.32023 (10)	0.3634 (2)	−0.04014 (5)	0.0563 (4)	
H26	0.3190	0.4453	−0.0652	0.068*	
C27	0.27864 (8)	0.0814 (2)	−0.01026 (4)	0.0462 (3)	
C28	0.23074 (8)	−0.1957 (2)	0.01966 (5)	0.0505 (3)	
H28	0.2013	−0.3052	0.0146	0.061*	
C29	0.36339 (8)	0.4072 (2)	0.00453 (5)	0.0485 (3)	
C30	0.32048 (7)	0.12731 (19)	0.03493 (4)	0.0421 (3)	
C31	0.26984 (8)	−0.14760 (19)	0.06465 (5)	0.0453 (3)	
C32	0.36395 (8)	0.29237 (19)	0.04120 (4)	0.0443 (3)	
H32	0.3934	0.3230	0.0710	0.053*	
C33	0.31488 (8)	0.00766 (19)	0.07226 (4)	0.0420 (3)	
C34	0.44996 (10)	0.6200 (2)	0.05015 (5)	0.0582 (4)	
H34A	0.4968	0.5373	0.0582	0.070*	
H34B	0.4139	0.6169	0.0745	0.070*	
H34C	0.4717	0.7409	0.0476	0.070*	
C35	0.21515 (10)	−0.4043 (2)	0.09906 (6)	0.0609 (4)	
H35A	0.1574	−0.3683	0.0876	0.073*	
H35B	0.2173	−0.4613	0.1296	0.073*	
H35C	0.2343	−0.4886	0.0771	0.073*	
C36	0.36123 (8)	0.04884 (19)	0.12026 (4)	0.0425 (3)	
C37	0.31181 (8)	0.10627 (17)	0.15718 (4)	0.0387 (3)	
C38	0.43097 (8)	0.0746 (2)	0.22378 (5)	0.0491 (3)	
H38	0.4649	0.0167	0.2042	0.059*	
C39	0.22936 (8)	0.15209 (18)	0.14364 (4)	0.0438 (3)	
H39	0.2047	0.1315	0.1123	0.053*	
C40	0.34895 (8)	0.12952 (18)	0.20514 (4)	0.0404 (3)	
C41	0.46189 (10)	0.1041 (3)	0.26970 (5)	0.0623 (4)	
H41	0.5167	0.0645	0.2818	0.075*	
C42	0.18033 (9)	0.2282 (2)	0.17458 (5)	0.0503 (3)	
H42	0.1233	0.2568	0.1643	0.060*	
C43	0.29889 (9)	0.21202 (19)	0.23588 (5)	0.0460 (3)	
C44	0.41358 (11)	0.1922 (3)	0.29922 (5)	0.0682 (5)	
H44	0.4367	0.2159	0.3307	0.082*	
C45	0.21523 (9)	0.26059 (19)	0.21939 (5)	0.0503 (3)	
H45	0.1828	0.3169	0.2400	0.060*	
C46	0.33410 (11)	0.2433 (2)	0.28285 (5)	0.0591 (4)	
H46	0.3016	0.3009	0.3032	0.071*	

### Atomic displacement parameters (Å^2^)

**Table:**

	*U*^11^	*U*^22^	*U*^33^	*U*^12^	*U*^13^	*U*^23^
O1	0.0597 (6)	0.0755 (8)	0.0473 (6)	0.0055 (5)	0.0005 (5)	−0.0061 (5)
O2	0.0410 (5)	0.0697 (7)	0.0426 (5)	−0.0012 (4)	0.0086 (4)	−0.0032 (4)
O3	0.0889 (8)	0.0381 (6)	0.0710 (7)	0.0088 (5)	0.0490 (6)	0.0040 (5)
O4	0.0575 (6)	0.0554 (6)	0.0525 (6)	−0.0099 (5)	0.0060 (4)	0.0040 (5)
O5	0.0605 (6)	0.0588 (7)	0.0532 (6)	0.0020 (5)	0.0145 (5)	0.0091 (5)
O6	0.0373 (5)	0.1113 (10)	0.0429 (5)	−0.0012 (5)	0.0053 (4)	0.0015 (6)
C1	0.0653 (9)	0.0448 (8)	0.0432 (7)	0.0004 (7)	0.0247 (6)	−0.0036 (6)
C2	0.0507 (7)	0.0416 (7)	0.0519 (8)	0.0010 (6)	0.0257 (6)	−0.0020 (6)
C3	0.0709 (9)	0.0524 (9)	0.0371 (7)	0.0003 (7)	0.0136 (6)	−0.0045 (6)
C4	0.0517 (7)	0.0344 (7)	0.0443 (7)	−0.0010 (5)	0.0208 (6)	−0.0022 (5)
C5	0.0410 (7)	0.0462 (8)	0.0559 (8)	0.0014 (6)	0.0164 (6)	−0.0001 (6)
C6	0.0553 (8)	0.0429 (8)	0.0432 (7)	−0.0019 (6)	0.0080 (6)	−0.0028 (6)
C7	0.0470 (7)	0.0308 (6)	0.0398 (6)	−0.0027 (5)	0.0158 (5)	−0.0017 (5)
C8	0.0449 (7)	0.0396 (7)	0.0408 (7)	−0.0013 (5)	0.0128 (5)	−0.0015 (5)
C9	0.0471 (7)	0.0385 (7)	0.0419 (7)	−0.0016 (5)	0.0142 (5)	−0.0037 (5)
C10	0.0424 (6)	0.0344 (7)	0.0394 (6)	−0.0020 (5)	0.0144 (5)	−0.0021 (5)
C11	0.0542 (9)	0.0850 (13)	0.0675 (10)	0.0114 (8)	−0.0017 (8)	−0.0125 (9)
C12	0.0455 (8)	0.0787 (11)	0.0542 (8)	0.0037 (7)	0.0036 (6)	0.0021 (8)
C13	0.0435 (6)	0.0381 (7)	0.0371 (6)	−0.0011 (5)	0.0121 (5)	−0.0043 (5)
C14	0.0335 (6)	0.0387 (7)	0.0318 (6)	−0.0007 (5)	0.0044 (4)	−0.0031 (5)
C15	0.0377 (6)	0.0428 (7)	0.0438 (7)	0.0009 (5)	0.0110 (5)	0.0004 (5)
C16	0.0428 (6)	0.0397 (7)	0.0415 (6)	0.0007 (5)	0.0112 (5)	−0.0053 (5)
C17	0.0301 (5)	0.0425 (7)	0.0302 (5)	−0.0020 (5)	0.0024 (4)	−0.0030 (5)
C18	0.0418 (7)	0.0504 (8)	0.0513 (7)	0.0047 (6)	0.0134 (6)	−0.0060 (6)
C19	0.0536 (8)	0.0363 (7)	0.0517 (7)	0.0027 (6)	0.0116 (6)	−0.0003 (6)
C20	0.0364 (6)	0.0449 (7)	0.0331 (6)	−0.0035 (5)	0.0031 (5)	−0.0006 (5)
C21	0.0376 (6)	0.0643 (9)	0.0409 (7)	−0.0009 (6)	0.0135 (5)	−0.0043 (6)
C22	0.0525 (7)	0.0410 (7)	0.0431 (7)	−0.0054 (6)	0.0078 (6)	0.0049 (6)
C23	0.0406 (6)	0.0550 (8)	0.0372 (6)	−0.0075 (6)	0.0079 (5)	0.0021 (6)
C24	0.0510 (8)	0.0819 (11)	0.0329 (7)	0.0076 (8)	0.0059 (6)	0.0000 (7)
C25	0.0404 (7)	0.0739 (10)	0.0397 (7)	0.0040 (7)	0.0051 (5)	−0.0139 (7)
C26	0.0573 (8)	0.0737 (11)	0.0394 (7)	0.0094 (8)	0.0117 (6)	0.0113 (7)
C27	0.0381 (6)	0.0671 (9)	0.0341 (6)	0.0077 (6)	0.0079 (5)	−0.0053 (6)
C28	0.0408 (7)	0.0597 (9)	0.0512 (8)	−0.0009 (6)	0.0074 (6)	−0.0134 (7)
C29	0.0444 (7)	0.0586 (9)	0.0449 (7)	0.0074 (6)	0.0142 (6)	0.0036 (6)
C30	0.0355 (6)	0.0566 (8)	0.0350 (6)	0.0065 (6)	0.0086 (5)	−0.0026 (6)
C31	0.0384 (6)	0.0543 (8)	0.0438 (7)	0.0026 (6)	0.0081 (5)	−0.0022 (6)
C32	0.0404 (6)	0.0573 (8)	0.0361 (6)	0.0038 (6)	0.0087 (5)	−0.0001 (6)
C33	0.0368 (6)	0.0528 (8)	0.0366 (6)	0.0027 (6)	0.0066 (5)	−0.0028 (6)
C34	0.0562 (8)	0.0581 (9)	0.0617 (9)	−0.0018 (7)	0.0130 (7)	0.0001 (7)
C35	0.0583 (9)	0.0548 (9)	0.0710 (10)	−0.0091 (7)	0.0144 (7)	−0.0016 (8)
C36	0.0386 (6)	0.0508 (8)	0.0376 (6)	−0.0023 (6)	0.0042 (5)	0.0052 (6)
C37	0.0401 (6)	0.0398 (7)	0.0358 (6)	−0.0048 (5)	0.0047 (5)	0.0021 (5)
C38	0.0453 (7)	0.0608 (9)	0.0399 (7)	−0.0078 (6)	0.0015 (5)	0.0063 (6)
C39	0.0428 (7)	0.0485 (8)	0.0393 (7)	−0.0003 (6)	0.0029 (5)	0.0009 (6)
C40	0.0436 (7)	0.0409 (7)	0.0363 (6)	−0.0085 (5)	0.0045 (5)	0.0025 (5)
C41	0.0531 (8)	0.0844 (12)	0.0452 (8)	−0.0132 (8)	−0.0063 (6)	0.0080 (8)
C42	0.0440 (7)	0.0530 (9)	0.0541 (8)	0.0053 (6)	0.0083 (6)	0.0008 (6)
C43	0.0554 (8)	0.0425 (8)	0.0408 (7)	−0.0099 (6)	0.0099 (6)	−0.0012 (6)
C44	0.0762 (11)	0.0868 (13)	0.0385 (8)	−0.0232 (9)	−0.0022 (7)	−0.0059 (8)
C45	0.0563 (8)	0.0458 (8)	0.0518 (8)	−0.0004 (6)	0.0180 (6)	−0.0033 (6)
C46	0.0749 (10)	0.0619 (10)	0.0409 (7)	−0.0145 (8)	0.0102 (7)	−0.0089 (7)

### Geometric parameters (Å, º)

**Table:**

O1—C6	1.3648 (17)	C20—C23	1.4196 (17)
O1—C11	1.4173 (19)	C21—C23	1.359 (2)
O2—C8	1.3690 (15)	C21—H21	0.9500
O2—C12	1.4232 (17)	C22—H22	0.9500
O3—C13	1.2185 (16)	C23—H23	0.9500
O4—C31	1.3723 (16)	C24—C26	1.356 (2)
O4—C35	1.4203 (18)	C24—C27	1.418 (2)
O5—C29	1.3669 (18)	C24—H24	0.9500
O5—C34	1.4283 (18)	C25—C28	1.371 (2)
O6—C36	1.2111 (15)	C25—C27	1.404 (2)
C1—C3	1.355 (2)	C25—H25	0.9500
C1—C4	1.4206 (19)	C26—C29	1.416 (2)
C1—H1	0.9500	C26—H26	0.9500
C2—C5	1.365 (2)	C27—C30	1.4250 (17)
C2—C4	1.405 (2)	C28—C31	1.4099 (19)
C2—H2	0.9500	C28—H28	0.9500
C3—C6	1.415 (2)	C29—C32	1.3686 (19)
C3—H3	0.9500	C30—C33	1.4208 (18)
C4—C7	1.4257 (17)	C30—C32	1.423 (2)
C5—C8	1.4104 (17)	C31—C33	1.3749 (19)
C5—H5	0.9500	C32—H32	0.9500
C6—C9	1.3718 (18)	C33—C36	1.5116 (17)
C7—C9	1.4163 (18)	C34—H34A	0.9800
C7—C10	1.4187 (17)	C34—H34B	0.9800
C8—C10	1.3767 (18)	C34—H34C	0.9800
C9—H9	0.9500	C35—H35A	0.9800
C10—C13	1.5106 (16)	C35—H35B	0.9800
C11—H11A	0.9800	C35—H35C	0.9800
C11—H11B	0.9800	C36—C37	1.4913 (18)
C11—H11C	0.9800	C37—C39	1.3745 (18)
C12—H12A	0.9800	C37—C40	1.4417 (17)
C12—H12B	0.9800	C38—C41	1.3694 (19)
C12—H12C	0.9800	C38—C40	1.4154 (18)
C13—C14	1.4904 (17)	C38—H38	0.9500
C14—C16	1.3772 (18)	C39—C42	1.4036 (19)
C14—C17	1.4457 (16)	C39—H39	0.9500
C15—C18	1.3742 (17)	C40—C43	1.4307 (19)
C15—C17	1.4148 (18)	C41—C44	1.406 (3)
C15—H15	0.9500	C41—H41	0.9500
C16—C19	1.4031 (19)	C42—C45	1.360 (2)
C16—H16	0.9500	C42—H42	0.9500
C17—C20	1.4226 (17)	C43—C45	1.412 (2)
C18—C21	1.397 (2)	C43—C46	1.4160 (19)
C18—H18	0.9500	C44—C46	1.356 (2)
C19—C22	1.3583 (19)	C44—H44	0.9500
C19—H19	0.9500	C45—H45	0.9500
C20—C22	1.4156 (19)	C46—H46	0.9500
			
C6—O1—C11	117.34 (11)	C21—C23—H23	119.3
C8—O2—C12	118.39 (10)	C20—C23—H23	119.3
C31—O4—C35	118.47 (12)	C26—C24—C27	121.61 (13)
C29—O5—C34	117.53 (11)	C26—C24—H24	119.2
C3—C1—C4	121.59 (12)	C27—C24—H24	119.2
C3—C1—H1	119.2	C28—C25—C27	121.89 (13)
C4—C1—H1	119.2	C28—C25—H25	119.1
C5—C2—C4	122.04 (12)	C27—C25—H25	119.1
C5—C2—H2	119.0	C24—C26—C29	119.94 (14)
C4—C2—H2	119.0	C24—C26—H26	120.0
C1—C3—C6	119.90 (12)	C29—C26—H26	120.0
C1—C3—H3	120.1	C25—C27—C24	122.30 (13)
C6—C3—H3	120.1	C25—C27—C30	119.30 (13)
C2—C4—C1	122.43 (12)	C24—C27—C30	118.39 (14)
C2—C4—C7	119.33 (12)	C25—C28—C31	118.77 (14)
C1—C4—C7	118.24 (12)	C25—C28—H28	120.6
C2—C5—C8	118.98 (12)	C31—C28—H28	120.6
C2—C5—H5	120.5	O5—C29—C32	125.16 (13)
C8—C5—H5	120.5	O5—C29—C26	114.14 (13)
O1—C6—C9	125.09 (13)	C32—C29—C26	120.70 (14)
O1—C6—C3	114.20 (12)	C33—C30—C32	122.67 (11)
C9—C6—C3	120.71 (13)	C33—C30—C27	118.22 (13)
C9—C7—C10	122.79 (11)	C32—C30—C27	119.08 (12)
C9—C7—C4	119.34 (11)	O4—C31—C33	115.45 (12)
C10—C7—C4	117.88 (12)	O4—C31—C28	123.21 (13)
O2—C8—C10	115.57 (11)	C33—C31—C28	121.32 (13)
O2—C8—C5	123.61 (12)	C29—C32—C30	120.23 (12)
C10—C8—C5	120.82 (12)	C29—C32—H32	119.9
C6—C9—C7	120.18 (12)	C30—C32—H32	119.9
C6—C9—H9	119.9	C31—C33—C30	120.42 (12)
C7—C9—H9	119.9	C31—C33—C36	119.95 (12)
C8—C10—C7	120.93 (11)	C30—C33—C36	119.60 (12)
C8—C10—C13	119.38 (11)	O5—C34—H34A	109.5
C7—C10—C13	119.54 (11)	O5—C34—H34B	109.5
O1—C11—H11A	109.5	H34A—C34—H34B	109.5
O1—C11—H11B	109.5	O5—C34—H34C	109.5
H11A—C11—H11B	109.5	H34A—C34—H34C	109.5
O1—C11—H11C	109.5	H34B—C34—H34C	109.5
H11A—C11—H11C	109.5	O4—C35—H35A	109.5
H11B—C11—H11C	109.5	O4—C35—H35B	109.5
O2—C12—H12A	109.5	H35A—C35—H35B	109.5
O2—C12—H12B	109.5	O4—C35—H35C	109.5
H12A—C12—H12B	109.5	H35A—C35—H35C	109.5
O2—C12—H12C	109.5	H35B—C35—H35C	109.5
H12A—C12—H12C	109.5	O6—C36—C37	122.90 (12)
H12B—C12—H12C	109.5	O6—C36—C33	118.52 (11)
O3—C13—C14	122.68 (11)	C37—C36—C33	118.44 (10)
O3—C13—C10	118.61 (11)	C39—C37—C40	119.45 (12)
C14—C13—C10	118.69 (11)	C39—C37—C36	117.78 (11)
C16—C14—C17	119.40 (11)	C40—C37—C36	122.55 (11)
C16—C14—C13	117.37 (10)	C41—C38—C40	120.63 (14)
C17—C14—C13	123.22 (11)	C41—C38—H38	119.7
C18—C15—C17	121.02 (12)	C40—C38—H38	119.7
C18—C15—H15	119.5	C37—C39—C42	122.25 (12)
C17—C15—H15	119.5	C37—C39—H39	118.9
C14—C16—C19	121.98 (11)	C42—C39—H39	118.9
C14—C16—H16	119.0	C38—C40—C43	118.12 (12)
C19—C16—H16	119.0	C38—C40—C37	124.39 (12)
C15—C17—C20	117.50 (10)	C43—C40—C37	117.49 (12)
C15—C17—C14	124.80 (11)	C38—C41—C44	120.90 (15)
C20—C17—C14	117.70 (11)	C38—C41—H41	119.6
C15—C18—C21	121.29 (13)	C44—C41—H41	119.6
C15—C18—H18	119.4	C45—C42—C39	119.43 (13)
C21—C18—H18	119.4	C45—C42—H42	120.3
C22—C19—C16	119.74 (12)	C39—C42—H42	120.3
C22—C19—H19	120.1	C45—C43—C46	120.67 (13)
C16—C19—H19	120.1	C45—C43—C40	120.09 (12)
C22—C20—C23	120.30 (12)	C46—C43—C40	119.24 (13)
C22—C20—C17	120.15 (11)	C46—C44—C41	120.11 (14)
C23—C20—C17	119.55 (12)	C46—C44—H44	119.9
C23—C21—C18	119.29 (11)	C41—C44—H44	119.9
C23—C21—H21	120.4	C42—C45—C43	121.14 (13)
C18—C21—H21	120.4	C42—C45—H45	119.4
C19—C22—C20	121.01 (12)	C43—C45—H45	119.4
C19—C22—H22	119.5	C44—C46—C43	120.91 (15)
C20—C22—H22	119.5	C44—C46—H46	119.5
C21—C23—C20	121.34 (12)	C43—C46—H46	119.5
			
C4—C1—C3—C6	−0.1 (2)	C27—C24—C26—C29	2.2 (2)
C5—C2—C4—C1	−179.99 (13)	C28—C25—C27—C24	−176.55 (13)
C5—C2—C4—C7	0.0 (2)	C28—C25—C27—C30	2.44 (19)
C3—C1—C4—C2	178.43 (14)	C26—C24—C27—C25	178.25 (13)
C3—C1—C4—C7	−1.5 (2)	C26—C24—C27—C30	−0.7 (2)
C4—C2—C5—C8	−1.2 (2)	C27—C25—C28—C31	−0.4 (2)
C11—O1—C6—C9	−1.3 (2)	C34—O5—C29—C32	2.10 (19)
C11—O1—C6—C3	178.34 (14)	C34—O5—C29—C26	−177.82 (12)
C1—C3—C6—O1	−177.64 (13)	C24—C26—C29—O5	178.30 (13)
C1—C3—C6—C9	2.0 (2)	C24—C26—C29—C32	−1.6 (2)
C2—C4—C7—C9	−178.59 (12)	C25—C27—C30—C33	−2.09 (18)
C1—C4—C7—C9	1.37 (18)	C24—C27—C30—C33	176.95 (12)
C2—C4—C7—C10	1.32 (18)	C25—C27—C30—C32	179.70 (11)
C1—C4—C7—C10	−178.72 (12)	C24—C27—C30—C32	−1.26 (18)
C12—O2—C8—C10	−179.74 (13)	C35—O4—C31—C33	173.24 (12)
C12—O2—C8—C5	0.4 (2)	C35—O4—C31—C28	−8.38 (19)
C2—C5—C8—O2	−178.97 (12)	C25—C28—C31—O4	179.66 (12)
C2—C5—C8—C10	1.2 (2)	C25—C28—C31—C33	−2.0 (2)
O1—C6—C9—C7	177.46 (13)	O5—C29—C32—C30	179.68 (12)
C3—C6—C9—C7	−2.1 (2)	C26—C29—C32—C30	−0.41 (19)
C10—C7—C9—C6	−179.49 (12)	C33—C30—C32—C29	−176.30 (12)
C4—C7—C9—C6	0.42 (19)	C27—C30—C32—C29	1.83 (18)
O2—C8—C10—C7	−179.73 (11)	O4—C31—C33—C30	−179.23 (11)
C5—C8—C10—C7	0.1 (2)	C28—C31—C33—C30	2.35 (19)
O2—C8—C10—C13	−4.04 (18)	O4—C31—C33—C36	2.86 (17)
C5—C8—C10—C13	175.77 (12)	C28—C31—C33—C36	−175.56 (12)
C9—C7—C10—C8	178.56 (12)	C32—C30—C33—C31	177.89 (12)
C4—C7—C10—C8	−1.35 (18)	C27—C30—C33—C31	−0.25 (18)
C9—C7—C10—C13	2.88 (18)	C32—C30—C33—C36	−4.19 (18)
C4—C7—C10—C13	−177.03 (11)	C27—C30—C33—C36	177.66 (11)
C8—C10—C13—O3	−102.32 (16)	C31—C33—C36—O6	109.71 (15)
C7—C10—C13—O3	73.42 (17)	C30—C33—C36—O6	−68.22 (18)
C8—C10—C13—C14	79.48 (15)	C31—C33—C36—C37	−74.50 (17)
C7—C10—C13—C14	−104.78 (13)	C30—C33—C36—C37	107.57 (14)
O3—C13—C14—C16	−175.74 (13)	O6—C36—C37—C39	163.18 (14)
C10—C13—C14—C16	2.38 (16)	C33—C36—C37—C39	−12.41 (18)
O3—C13—C14—C17	3.47 (19)	O6—C36—C37—C40	−11.4 (2)
C10—C13—C14—C17	−178.41 (11)	C33—C36—C37—C40	172.99 (12)
C17—C14—C16—C19	0.23 (18)	C40—C37—C39—C42	2.7 (2)
C13—C14—C16—C19	179.47 (11)	C36—C37—C39—C42	−172.09 (13)
C18—C15—C17—C20	−0.06 (18)	C41—C38—C40—C43	−1.6 (2)
C18—C15—C17—C14	179.79 (11)	C41—C38—C40—C37	179.08 (13)
C16—C14—C17—C15	−179.70 (11)	C39—C37—C40—C38	174.95 (13)
C13—C14—C17—C15	1.10 (18)	C36—C37—C40—C38	−10.5 (2)
C16—C14—C17—C20	0.15 (16)	C39—C37—C40—C43	−4.35 (19)
C13—C14—C17—C20	−179.05 (10)	C36—C37—C40—C43	170.16 (12)
C17—C15—C18—C21	0.9 (2)	C40—C38—C41—C44	−1.1 (2)
C14—C16—C19—C22	−0.2 (2)	C37—C39—C42—C45	0.9 (2)
C15—C17—C20—C22	179.35 (11)	C38—C40—C43—C45	−176.72 (13)
C14—C17—C20—C22	−0.51 (16)	C37—C40—C43—C45	2.63 (19)
C15—C17—C20—C23	−0.84 (16)	C38—C40—C43—C46	2.97 (19)
C14—C17—C20—C23	179.30 (10)	C37—C40—C43—C46	−177.68 (12)
C15—C18—C21—C23	−0.9 (2)	C38—C41—C44—C46	2.5 (3)
C16—C19—C22—C20	−0.1 (2)	C39—C42—C45—C43	−2.7 (2)
C23—C20—C22—C19	−179.29 (12)	C46—C43—C45—C42	−178.78 (14)
C17—C20—C22—C19	0.52 (19)	C40—C43—C45—C42	0.9 (2)
C18—C21—C23—C20	−0.06 (19)	C41—C44—C46—C43	−1.1 (3)
C22—C20—C23—C21	−179.27 (12)	C45—C43—C46—C44	178.04 (15)
C17—C20—C23—C21	0.92 (18)	C40—C43—C46—C44	−1.7 (2)

### Hydrogen-bond geometry (Å, º)

**Table:**

*D*—H···*A*	*D*—H	H···*A*	*D*···*A*	*D*—H···*A*
C12—H12*B*···O6	0.98	2.51	3.2862 (18)	136
C19—H19···O3^i^	0.95	2.40	3.2418 (17)	148
C15—H15···O3	0.95	2.19	2.8397 (16)	125
C38—H38···O6	0.95	2.25	2.8548 (17)	121

Symmetry code: (i) *x*, *y*+1, *z*.
